# Cases Which May Benefit from Surgical Measures

**Published:** 1919-08-16

**Authors:** 


### THE TREATMENT OF ASTHMA.

### Cases which may Benefit from Surgical Measures.

This disease has received an enormous amount of attention from writers, and judging from many of their reports, one would be inclined to imagine that the cure was fairly simple; actually it is certain that we have not yet solved the mystery to anything like its full extent. We do not think that any surgeon can honestly guarantee a cure, but he can certainly promise great relief in some cases, and although space will not permit of a discussion on the detailed pathology of this condition, yet it is hoped that the following notes will assist the practitioner in deciding which cases are likely to benefit from surgical interference.

In every case of asthma the nose, naso-pharynx, mouth, and larynx should be examined, preferably in that order, and one should always be prepared to examine the frontal, maxillary, and sphenoidal sinuses should any suspicion arise during the nasal examination of their being unhealthy.

### Clinical Conditions.

The following lesions may be found:

(*a*) *Nose.*—(1) Septum markedly deflected to R. or L., or both, giving rise to variable but definite degrees of nasal obstruction; (2) septal spur, often causing pressure against either inferior or middle turbinates; (3) true hypertrophic rhinitis, with definite hyperplasia often extending to the bone; (4) transient hypertrophic rhinitis, vasomotor in origin, which may affect either the inferior or middle turbinates, more often the former; (5) polypoid ethmoiditis, without apparent suppuration; (6) suppurating ethmoiditis.

In these two last conditions the maxillary and frontal sinuses should be carefully inspected, otherwise treatment directed only to the apparent lesion in the ethmoid may fail to relieve, and therefore weaken the patient's faith in the treatment, a most important item in the treatment of neuroses, a partial example of which the asthmatic condition so frequently forms. Also it must be noted that when there is a marked area of hyperæsthesia on the septum or turbinates, great benefit can often be derived from judicious cauterisation.

(*b*) *Naso-Pharynx.*—Here may be found diseased adenoids or polypoid growths.

(*c*) *Mouth.*—Inspection may at once show diseased tonsils or diseased teeth, and both these lesions have a profound influence on the whole respiratory tract.

(*d*) *Larynx.*—A foreign body, or irritation from an early growth, or chronic laryngitis may be an existing cause, but apart from such definite clinical conditions it must be remembered that any substance, such as pollen, dust, tobacco, etc., that tends to irritate the delicate mucous membrane in any part of the respiratory tract, predisposes at once to an asthmatic neurosis. Treatment then becomes largely preventive, but in all the classes under *a*, *b*, *c*, *d*, surgical treatment is scientifically rational, and in a great number it ensures great benefit and sometimes permanent cure.

